# Genomic-inferred cross-selection methods for multi-trait improvement in a recurrent selection breeding program

**DOI:** 10.1186/s13007-024-01258-4

**Published:** 2024-09-02

**Authors:** Sikiru Adeniyi Atanda, Nonoy Bandillo

**Affiliations:** 1 Agricultural Data Analytics Unit, North Dakota State University, Fargo, ND 58105-6050 USA; 2 Department of Plant Sciences, North Dakota State University, Fargo, ND 58108-6050 USA

**Keywords:** Selection index, Usefulness criterion, Genomic prediction, Genomic estimated breeding value, Optimal haploid value, Genetic gain, Genetic drift, Stochastic simulation, Quantitative trait nucleotide, Breeding cycle

## Abstract

The major drawback to the implementation of genomic selection in a breeding program lies in long-term decrease in additive genetic variance, which is a trade-off for rapid genetic improvement in short term. Balancing increase in genetic gain with retention of additive genetic variance necessitates careful optimization of this trade-off. In this study, we proposed an integrated index selection approach within the genomic inferred cross-selection (GCS) framework to maximize genetic gain across multiple traits. With this method, we identified optimal crosses that simultaneously maximize progeny performance and maintain genetic variance for multiple traits. Using a stochastic simulated recurrent breeding program over a 40-years period, we evaluated different GCS methods along with other factors, such as the number of parents, crosses, and progeny per cross, that influence genetic gain in a pulse crop breeding program. Across all breeding scenarios, the posterior mean variance consistently enhances genetic gain when compared to other methods, such as the usefulness criterion, optimal haploid value, mean genomic estimated breeding value, and mean index selection value of the superior parents. In addition, we provide a detailed strategy to optimize the number of parents, crosses, and progeny per cross that can potentially maximize short- and long-term genetic gain in a public breeding program.

**Supplementary Information:**

The online version contains supplementary material available at 10.1186/s13007-024-01258-4.

## Background

**Fig. 1:**
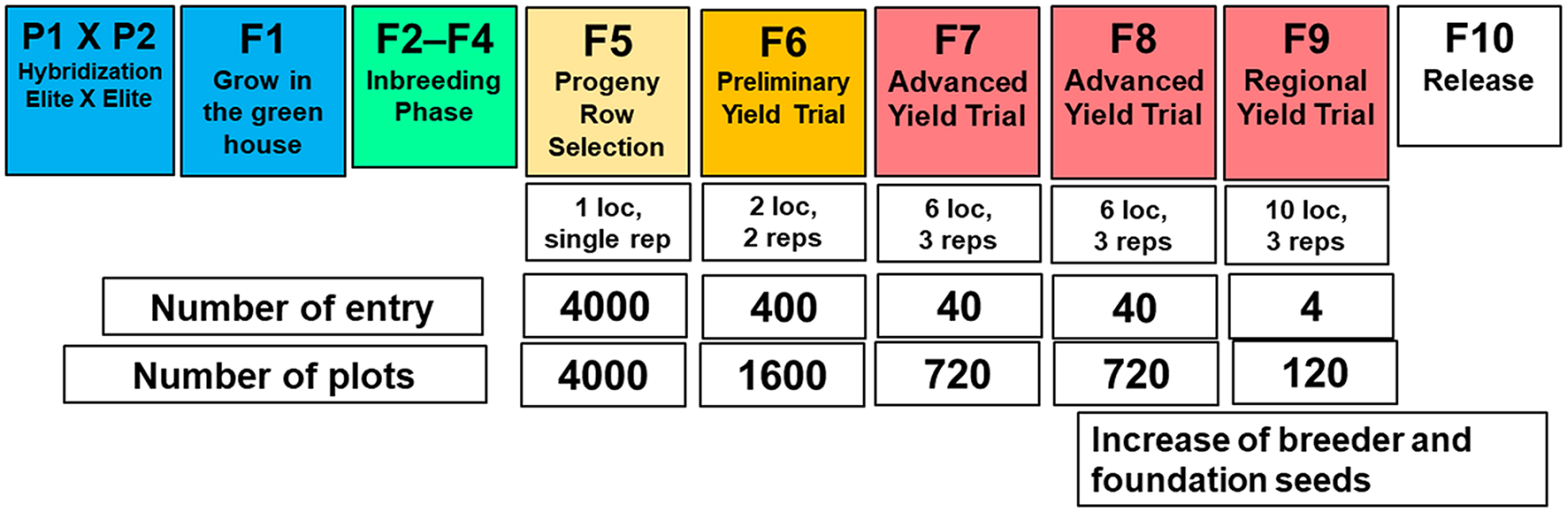
Schematic representation of the North Dakota State University pulse crop breeding program pipeline

Feeding the increasing world population requires doubling the current food production [1]. Achieving this goal requires accelerating genetic gain within the constraints of a limited budget and resources. The conventional selfing breeding scheme involves (i) parental selection for crossing to develop families; (ii) creating homogeneous progeny within families through selfing or double haploid technology; (iii) evaluating families in the nursery for morpho-agronomic and disease assessment; and (iv) advancing selected superior genotypes through the yield testing stages (Fig. 1). In general, the scheme is considered time-consuming, taking several evaluation stages for breeding materials to be recycled as parents and new varieties released as products [2, 3]. Reducing the breeding cycle time (the duration of time required to select parents back into the crossing block to create the next generation of families) has been identified as a key factor to further accelerate genetic gain [4–7].

Advancements in genotyping technology, decreasing associated costs [8, 9], and advances in statistical modeling and computing power have spurred the widespread adoption of genomic selection (GS) [2, 10, 11]. GS utilizes DNA information to predict the genomic estimated breeding values (GEBV) of new untested genotypes. It has been shown to be an innovative tool for reducing breeding cycle time and phenotyping expenses [2, 3, 5, 8, 12–14]. The acceleration in genetic progress is attributed to its ability to identify superior parent genotypes for breeding at an earlier stage compared to conventional phenotypic selection [5, 8, 12, 14]. However, the swift short-term genetic gain achieved through GS contributes to a faster reduction in genetic diversity in subsequent generations due to increased inbreeding [3, 4, 6, 15–17]. The primary determinant of prediction accuracy in GS relies on the genetic relatedness between the training and testing sets [8, 18, 19]. In other words, the superior genotypes selected through truncation selection are more likely to exhibit higher similarity due to the increased level of coancestry, resulting in higher inbreeding rates in each selection cycle. Several studies [12, 20–24] have suggested an alternative approach for sustainable genetic gain over both the short and long term in a plant breeding program. In contrast to interbreeding genotypes with the highest GEBVs (as in truncation selection), these strategies propose establishing crosses between genotypes based on a cross predicted usefulness or merit. Cross usefulness is a metric that optimizes the mean of the progeny and genetic variance within the bi-parental population (progeny that share the same parents from a single cross) [25, 26]. An example of a cross-selection method is the optimal haploid value (OHV) proposed by Daetwyler et al. [22]. OHV aims to maximize haplotype complementarity of the crossing parents. However, a limitation of OHV was the inability to consider linkage disequilibrium between quantitative trait loci (QTLs) and the complexity associated with optimally partitioning the genome into predefined haplotype segments [21, 27]. In another study, Lehermeier et al. [21] proposed a novel deterministic approach to predict the additive progeny variance of a cross from the phenotypic and genotypic information of the parents. The predicted additive progeny variance was used within the statistical framework of the usefulness criterion (UC) proposed by Schnell and Utz [25] to select parent combinations for crossing blocks. In general, these methods and others are typically evaluated based on individual traits. However, in practice, potential parents often possess multiple traits of economic and agronomic significance [28, 29]. These traits have attributes linked to productive performance, adaptability, and production stability. To improve multiple traits simultaneously, selection index methods are commonly employed. These methods combine all relevant traits into a single index and prove to be highly useful for improving multiple traits with the desired selection response [28, 30–32].

The Smith-Hazel selection index, which integrates genetic correlation with economic weights, has gained wide traction in animal breeding [28, 29, 34]. Determining suitable weights for different agronomic and quality traits remains a significant challenge, limiting the widespread adoption of this index in plant breeding. In this study, we consider the non-parametric rank summation index proposed by Mulamba and Mock [33]. It offers the distinct advantage of not requiring economic weights to compute the index for different genotypes [34–36]. The rank summation index is based on the ranking of genotypes in relation to the target trait and summing up the ranks for multiple traits simultaneously [33, 36–38]. Theoretically, selection on this index (a hypothetical new phenotype) should result in simultaneous improvements across all desired traits. Chung and Liao [39] similarly used a selection index to select individuals based on GEBVs for multiple traits. However, their strategy may inadvertently favor selection of parent combinations that share identical beneficial alleles, accelerating the loss of genetic diversity. To our knowledge, this is the first time an index selection (IS) will be utilized within the framework of GP to select genotypes and parental combinations for crossing blocks. Our objective aligns with Wolfe et al. [40], but our approach differs significantly. Wolfe et al. [40] suggested constructing a complete matrix of genetic variances and covariances for traits, a method that is computationally demanding and prone to model convergence issues, especially with small datasets and numerous traits. In contrast, our approach offers a less computationally intensive alternative to directly predict the variance of IS (new phenotype). Additionally, this study aims to identify the optimal number of parents, crosses, and progeny per cross in the North Dakota State University (NDSU) pulse crop breeding program using stochastic genetic simulation in the R package AlphaSimR [41].

## Materials and methods

### Founder population and genetic parameters

A pea (*Pisum sativum* L.) genome size (cM) and chromosome sizes described in [42] were simulated using the Markovian Coalescent Simulator (MaCS) [43] implemented in AlphasimR [41]. This resulted in a founder population of 200 non-inbred individuals with 7 chromosome pairs each.

In the base population, we assumed that 2,100 segregating sites were evenly distributed across the chromosomes. From these sites, we randomly sampled between 71 and 72 segregating sites per chromosome to serve as quantitative trait nucleotides (QTN), totaling 500 QTN. Additionally, we simulated SNP chip with 500 SNPs per chromosome for genotyping, resulting in a total of 3,500 single-nucleotide polymorphisms (SNPs). We simulated four polygenic traits: grain yield (YLD), 1000 kernel weight (TKW), days to physiological maturity (DPM), and plant height (PH). In quantitative genetic theory, it is assumed that the number of segregating QTNs for polygenic traits will exceed the number of independent chromosome segments (*M*_*e*_) [34, 44]. Pea has a long-range LD due to its selfing nature [45], and it presumably has *M*_*e*_ less than the 500 random QTN selected in our study. This aligns with several simulation studies that predominantly assume polygenic traits are controlled by 500 or greater QTN [46–50].

Each QTN was assigned an additive effect that was sampled from a Gaussian distribution with a mean and variance obtained from variance components estimated from a multivariate model fitted to NDSU historical field yield trials. The means were (YLD = 5.78, TKW = 433.00, DPM = 81.00, PH = 67.00), and the variances were (YLD = 3.59, TKW = 50.10, DPM = 12.24, PH = 15.80). For simplicity, similar to [50], we also omitted dominance and epistasis effects in the simulation.

### Phenotype simulation

Random noise sampled from a normal distribution with a mean of 0 and the error variance for the traits were added to the genetic values of the founder lines to produce the phenotype. The error variances were varied to reflect the plot-level heritability currently obtained in the breeding program for the yield testing stages. Entry-mean narrow-sense heritability was set to 0.1 in the nursery stage for visual selection, similar to Gaynor et al. [41]. The genetic correlation between traits (off-diagonal element) and broad-sense heritability (diagonal element) describing the genetic architecture of the traits are presented in (Supp. 1). The genotype-by-environment variance provided a non-heritable variation attributed to the locations; see Gaynor et al. [41] for detailed implementation in AlphaSimR.

### Simulation parameters

The simulation was based on an already established breeding pipeline of the NDSU pulse crop breeding program with several simulated treatments (Table 1). The selected parameters were determined mainly based on available breeding materials, power for making inferences, available resources, and practical relevance for successful implementation of GS in the NDSU elite breeding pipeline. In all treatment scenarios, the number of individuals in the F_2_ generation was restricted to 15,000, while in the progeny row or nursery, the limit was set at 4,000. We developed a grid to evaluate different numbers of parents: 30, 40, and 50 and 50, 100, 150, and 200 crosses, respectively. The number of progeny per cross was limited to 300, 150, 100, and 75, respectively. Thus, the number of F_2_ individuals (15,000), which is the number of crosses multiplied by the number of progeny per cross, is constant across treatments. We employed several methods to select parent pairings for crossing, including UC, Posterior Mean-Variance (PMV), OHV, Mean GEBV, and random mating of superior parents (denoted as RandPheno). In total, 60 simulation treatments were examined.

**Table 1:** Summary of the combination of the number of parents, crosses and progeny per cross used for the simulation study

Number of parents	Number of crosses	Progeny per cross
**30**	50	300
	100	150
	150	100
	200	75
**40**	50	300
	100	150
	150	100
	200	75
**50**	50	300
	100	150
	150	100
	200	75

*Note* Multiplying the number of unique combinations, which is 12, by the number of cross-selection metrics (UC, OHV, PMV, MeanGEBV and RandPheno) yields a total of 60 treatments

### Simulation scenario

We utilized the same base population across all treatments or breeding scenarios. Identification of superior genotypes as parents was performed based on the rank summation index (RSI) [33]. This approach entailed converting genotypic values into ranks, reflecting each genotype’s relative performance across multiple traits crucial to the breeding program’s objectives. This process involves transforming genotypic values into ranks, with the aim of either enhancing or achieving the optimal mean value for each desired trait. Subsequently, the ranks for each genotype across all selected traits were summed to compute the RSI and lower sums indicate better overall performance. This index serves as a holistic measure of genetic merit, enabling the identification of the most promising genotypes for further breeding efforts. For parent selection at the Preliminary Yield Trial (PYT) to initiate a new breeding cycle, the RSI ($\:{\text{y}}_{\text{I}\text{S}})$ for each genotype was computed as follows:

1 $${{\rm{y}}_{{\rm{IS}}}}\;{\rm{ = }}\;{\rm{\Sigma }}_{{\rm{j = 1}}}^{\rm{n}}\;{{\rm{g}}_{{\rm{ij}}}}\;$$

Each genotype was ranked based on its performance for each trait (n), where $\:{\text{g}}_{\text{i}\text{j}}$ is the rank of the i-th genotype for j-th trait and $\:{\text{y}}_{\text{I}\text{S}}$ is the sum of these ranks across all traits for each genotype. Therefore, aggregate performance information across multiple traits according to their significance and underlying genetic architecture. This aggregated index ($\:{\text{y}}_{\text{I}\text{S}}$) offers a comprehensive evaluation of genetic merit, capturing a broader genetic signal than could be obtained from any single trait. Although derived from ranks rather than direct measurements, $\:{\text{y}}_{\text{I}\text{S}}$ effectively assesses the composite genetic potential across multiple quantitative traits. Given that each contributing trait is influenced by numerous genes (polygenic nature), the aggregate genetic architecture influencing $\:{\text{y}}_{\text{I}\text{S}}$ variations is complex enough to warrant treating it as a quantitative trait. This approach, as applied in our study, facilitates a nuanced selection process, prioritizing genotypes with the highest composite genetic value as parents for next generation.

Using only 3,500 non-QTN markers, we fitted a whole-genome regression model (Eq. 2) using the derived phenotype ($\:{\text{y}}_{\text{I}\text{S}}$) as a response variable. We calculated Rogers’ distance based on marker data between all possible combinations of the selected parents. When mean GEBV of the parents were considered as method for parent pairing, only parental combinations with a genetic distance less than 0.1 were preselected before mean of the GEBV (MeanGEBV) was used as final decision method. This step was necessary to best simulate a typical procedure in a breeding program. For the UC, PMV, OHV and random crossing of the superior parents there was no prior selection of crosses.

2 $${{\rm{y}}_{{\rm{IS}}}}\;{\rm{ = }}\;{\rm{\mu }}\;{\rm{ + }}\;{\rm{\Sigma }}_{{\rm{j = 1}}}^{\rm{p}}\;{{\rm{X}}_{{\rm{ij}}}}\;{{\rm{\beta }}_{\rm{j}}}\;{\rm{ + }}\;{\rm{\varepsilon }}$$

where p is the marker size, $\bf \bf \bf \:{\mathbf{X}}_{\text{i}\text{j}}$ represent allele dosage at the j-th locus/QTN of the genome for the i-th line: 0 is the homozygous copies of the allele, 1 is the heterozygous copies of the allele, and 2 is the homozygous copies of the second allele, and $\it \it \it \it \it \it {{{\upbeta }}_{\rm{j}}}$ is the effect of marker j-th on $\:{\mathbf{y}}_{\text{I}\text{S}}.\:$ The marker effect was assumed to be independent and identical with Gaussian distributions ${\rm{\beta }}$~N(0, **I**${{\upsigma }}_{{\upbeta }}^{\rm{2}}$). Additionally, the residual error was assumed to be independent and identical with Gaussian distributions ${\rm{\varepsilon }}$~N(0, **I**${\rm{\sigma }}_{\rm{\varepsilon }}^{\rm{2}}$). The whole-genome regression model was fitted with Bayesian Ridge Regression implemented in the BGLR package [51]. This assumed scaled inverse-$\:{X}^{2}$ prior distributions assigned to the marker effects and residual variance (${\rm{\sigma }}_{\rm{\beta }}^{\rm{2}}$ and $]{\rm{\sigma }}_{\rm{\varepsilon }}^{\rm{2}}$), respectively. Samples from the posterior distribution were generated using the Markov chain Monte Carlo (MCMC) algorithm implemented in the BGLR package. We used 40,000 iterations, discarded the first 10,000 as burn-in and thinned to every 10th sample.

The estimated GEBV ($\:\widehat{{\mathbf{y}}_{\text{I}\text{S}}}$) is the product of estimated marker effects $\widehat {{{{\upbeta }}_{\rm{j}}}}$and allele dosages.

3 $${\rm{G}}\widehat {{\rm{EB}}{{\rm{V}}_{{\rm{IS}}}}}\;{\rm{ = }}\;{{\rm{X}}_{{\rm{ij}}}}\;\widehat {{{\rm{\beta }}_{\;{\rm{j}}}}}$$

The mean GEBV was obtained as follows:

4 $${\rm{\mu }}_{{\text{P}}_{{\text{A}}_{\text{I}\text{S}}}\text{x}{\text{P}}_{{\text{A}}_{\text{I}\text{S}}}}=\frac{1}{2}\left(\widehat{{\mathbf{G}\mathbf{E}\mathbf{B}\mathbf{V}}_{{\text{I}\text{S}}_{\text{A}}}}\:+\:\:\widehat{{\mathbf{G}\mathbf{E}\mathbf{B}\mathbf{V}}_{{\text{I}\text{S}}_{\text{B}}}}\:\right)$$

The formula for calculating PMV which is the expected variance of progeny for each P_Ax_P_B_  combination was formulated as proposed by Lehermeier et al. [21]:

5 $$\:{\text{P}\text{M}\text{V}}_{{\text{P}}_{{\text{A}}_{\text{I}\text{S}}}\text{x}{\text{P}}_{{\text{B}}_{\text{I}\text{S}}}}=\frac{1}{\text{L}}{\sum\:}_{\text{j}=1}^{\text{L}}{\rm{\beta }}^{{\left(\mathbf{j}\right)}^{\mathbf{{\prime\:}}}}\varvec{\Sigma\:}{\rm{\beta }}^{\left(\text{j}\right)}$$

L is the size of the posterior sample postburn-in, ${\rm{\beta }}^{\left(\text{j}\right)}$ is the j-th thinned postburn-in sample of the MCMC algorithm from the whole-genome regression model (Eq. 2) and $\:\varvec{\Sigma\:}\:$is the variance covariance matrix between DH parents $\:{\text{P}}_{\text{A}}x{\text{P}}_{\text{B}}$ alleles at QTN in progeny; see Lehermeier et al. [21] for details.

6 $$\:{\varvec{\Sigma\:}}_{\text{j}\text{k}}=4{\text{D}}_{\text{j}\text{k}}\text{*}\left(1-2{\text{c}}_{\text{j}\text{k}}^{\left(1\right)}\right)$$

$\:{\text{D}}_{\text{i}\text{j}}$ is the linkage disequilibrium (LD) parameter between alleles at loci j and k for parents $\:{\text{P}}_{\text{A}}x{\text{P}}_{\text{B}}$.

The parameter $\:{\text{D}}_{\text{i}\text{j}}$ would be 0 if both parental pairs share the same allele at either locus j or k. Alternatively, it assumed a value of 0.25 or -0.25, depending on the linkage phase of the parental pair. $\:{\text{c}}_{\text{j}\text{k}}$is the recombination rate between parental locus j and k. The recombination frequency was estimated using the genetic map information as follows:

7 $$\:{\text{c}}_{\text{j}\text{k}}=0.5\left(1-{\text{e}}^{{2\text{d}}_{\text{j}\text{k}}}\right)$$

where $\:{\text{d}}_{\text{j}\text{k}}$ is the map distance in morgan (M) between loci j and k [52].

In addition, the UC was estimated as follows:

8 $${\rm{U}}{{\rm{C}}_{{\rm{IS}}}}\; = \;{{{\upmu }}_{{\rm{IS}}}}\;{\rm{ + }}\;{\rm{i}}{{{\upsigma }}_{{{\rm{g}}_{{\rm{IS}}}}}}$$

where ${{{\upmu }}_{{\rm{IS}}}}$ is the mean of the genetic value of the cross, i is the selection intensity, and ${{{\upsigma }}_{{{\rm{g}}_{{\rm{IS}}}}}}$is the standard deviation estimated from Eq. 5. We calculated the standardized selection intensity using the following method in the R environment [53]:

9 $$\:\text{i}=\:\text{d}\text{n}\text{o}\text{r}\text{m}\left(\text{q}\text{n}\text{o}\text{r}\text{m}\left(1-\text{p}\right)\right)/\text{p}$$

where p is the selected proportion.

To obtain the OHV of the parental combination, it was estimated as follows:

10 $$\widehat {{\rm{OH}}{{\rm{V}}_{{\rm{IS}}}}}\;{\rm{ = }}\;{\rm{2\Sigma }}_{{\rm{j = 1}}}^{^{\rm{n}}{\rm{Segments}}}\;{\rm{max}}\;{\rm{(}}{{\rm{H}}^{\rm{j}}}{\widehat {{{\rm{\beta }}_{{\rm{IS}}}}}^{\rm{J}}}{\rm{)}}$$

where $\:{\text{n}}_{\text{S}\text{e}\text{g}\text{m}\text{e}\text{n}\text{t}\text{s}}$ is the number of segments into which the genome is split, $\:{\mathbf{H}}^{\text{j}}$ is the matrix containing the four haplotype scores (0 or 1) of the two parental lines, and ${\rm{\beta }}_{{\rm{IS}}}^{\rm{j}}$is the vector of marker effects of segment j estimated via Eq. 2 using the training population. See Daetwyler et al. [22] for details.

For all treatments, DH lines were made from the F_1_ to reduce computation time, and 15,000 individuals were generated for evaluation in the nursery. Visual selection with a heritability of 0.1 was assumed across traits following Gaynor et al. [41]. In the PYT, we evaluated 400 genotypes advanced from the nursery stage. These genotypes were evaluated for the four traits in two replicates across two locations. Based on the RSI, we identified superior genotypes and reintroduced them into the crossing block as parents to start new cycle. Cross combinations that generate progeny for the subsequent generations were determined using the different cross-selection methods.

We further narrowed the pool to the 40 most favorable genotypes for the advanced yield trials. These 40 genotypes were evaluated in three replicates across six locations and two years, providing us with comprehensive data on their performance for release as a variety.

Each treatment is independent, and the simulated breeding program spanned 40 years with a burn-in period of 10 years. Data regarding population mean, genetic gain, and genetic variance were collected for the 10 to 40 years of the simulation, which was represented as 0 to 30 in the study. Each simulation treatment was replicated 50 times.

## Results

### Efficiency of multi-trait genomic inferred cross-selection methods to simultaneously improve response to selection

**Fig. 2:**
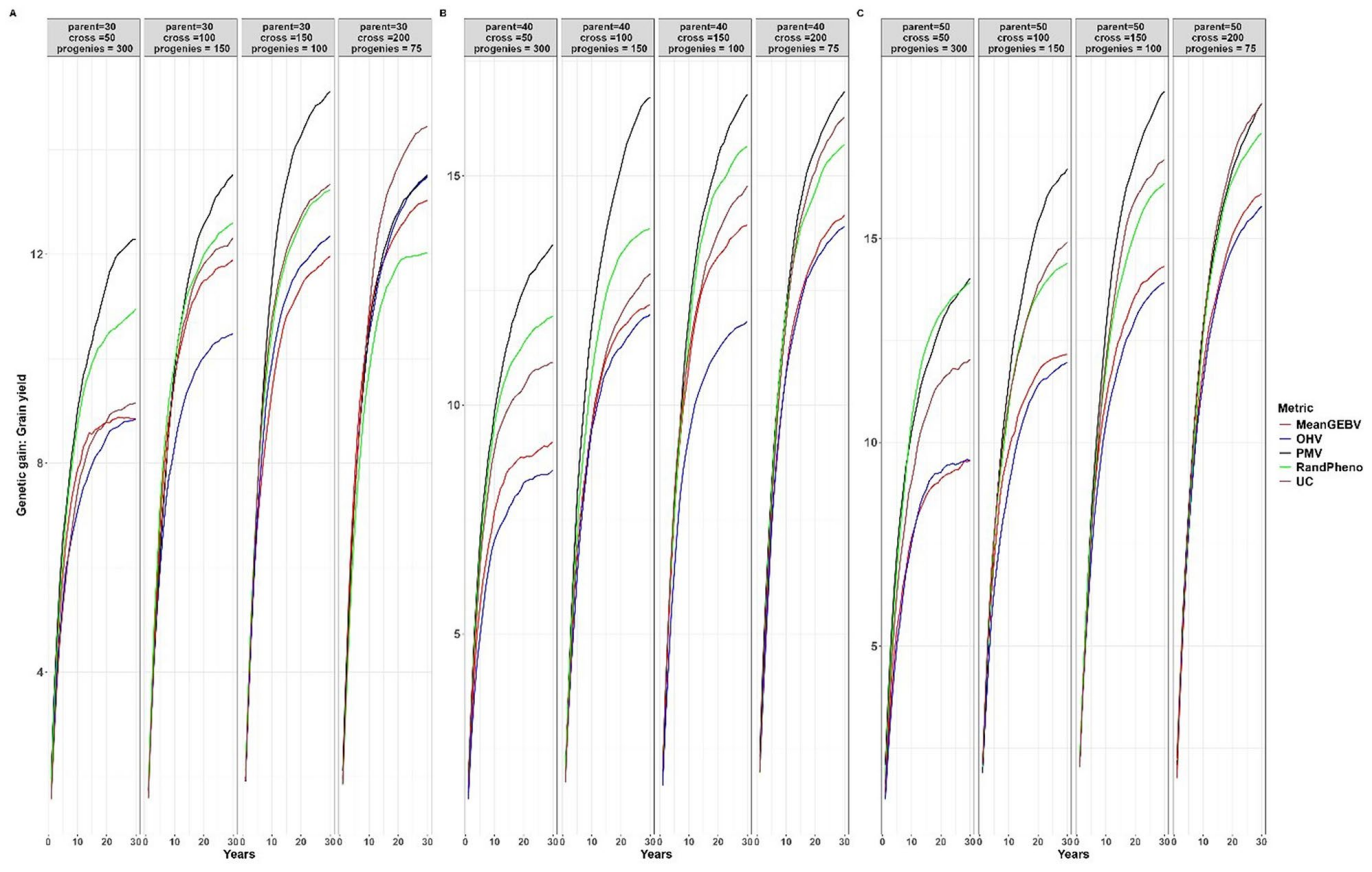
Genetic gains for different cross-selection methods and different numbers of parents, crosses and progeny per cross for grain yield over 30 years post burn-in. The red line (MeanGEBV) highlights the genetic gain obtained using the mean of the GEBV of the distantly related superior parents to select crosses, the green line (RandPheno) represents the random mating of the superior genotypes, the blue line (OHV) is the optimal haploid value, the black line (PMV) represents the posterior mean variance and the brown line (UC) represents the genetic gain observed using the usefulness criterion as a cross-selection metric

**Fig. 3:**
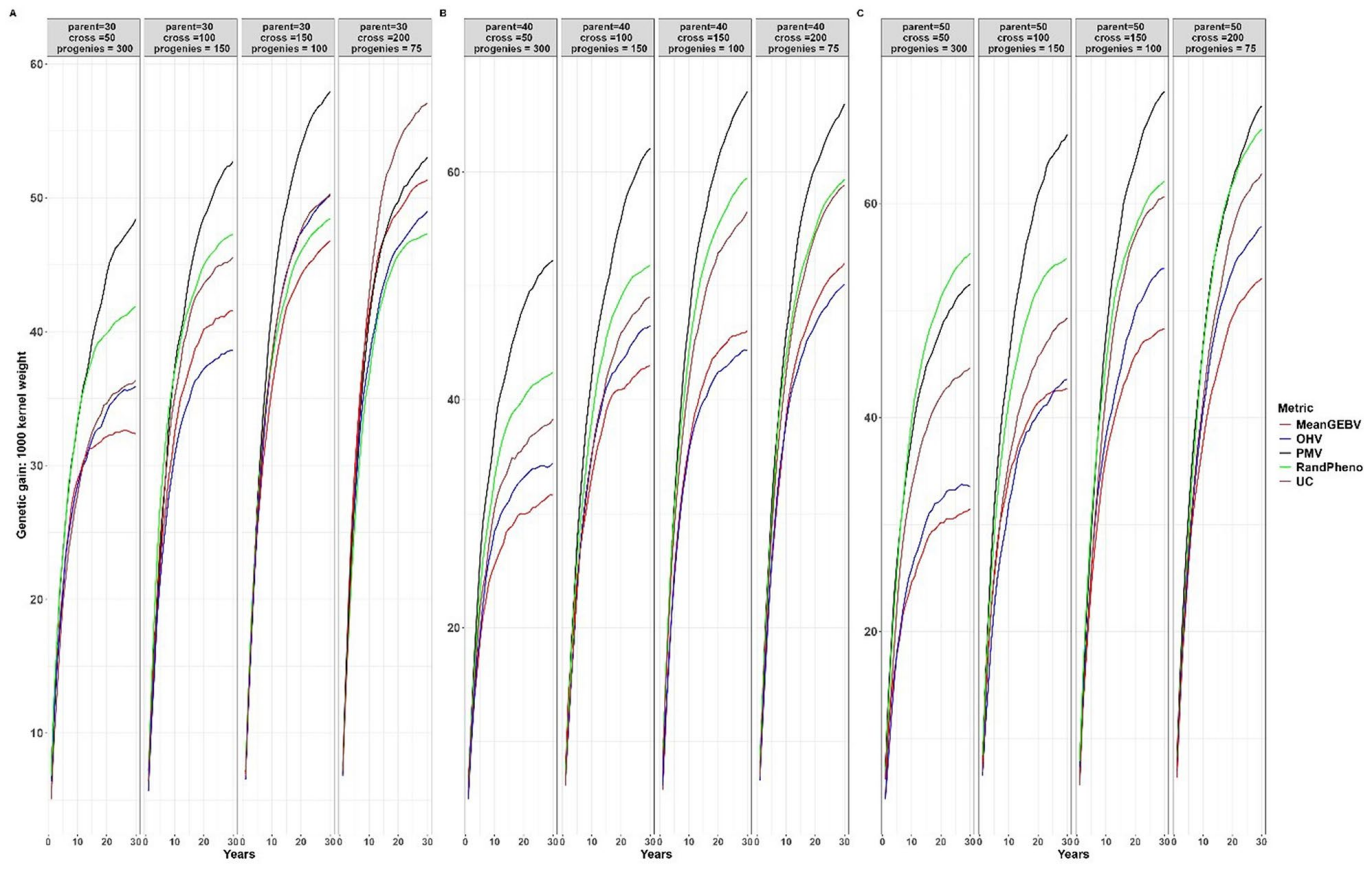
Genetic gains for different cross-selection methods and different numbers of parents, crosses and progeny per cross for 1000 kernel weight over 30 years post burn-in. The red line (MeanGEBV) highlights the genetic gain obtained using the mean of the GEBV of the distantly related superior parents to select crosses, the green line (RandPheno) represents the random mating of the superior genotypes, the blue line (OHV) is the optimal haploid value, the black line (PMV) represents the posterior mean variance and the brown line (UC) corresponds to the genetic gain observed using the usefulness criterion as a cross-selection metric

**Fig. 4:**
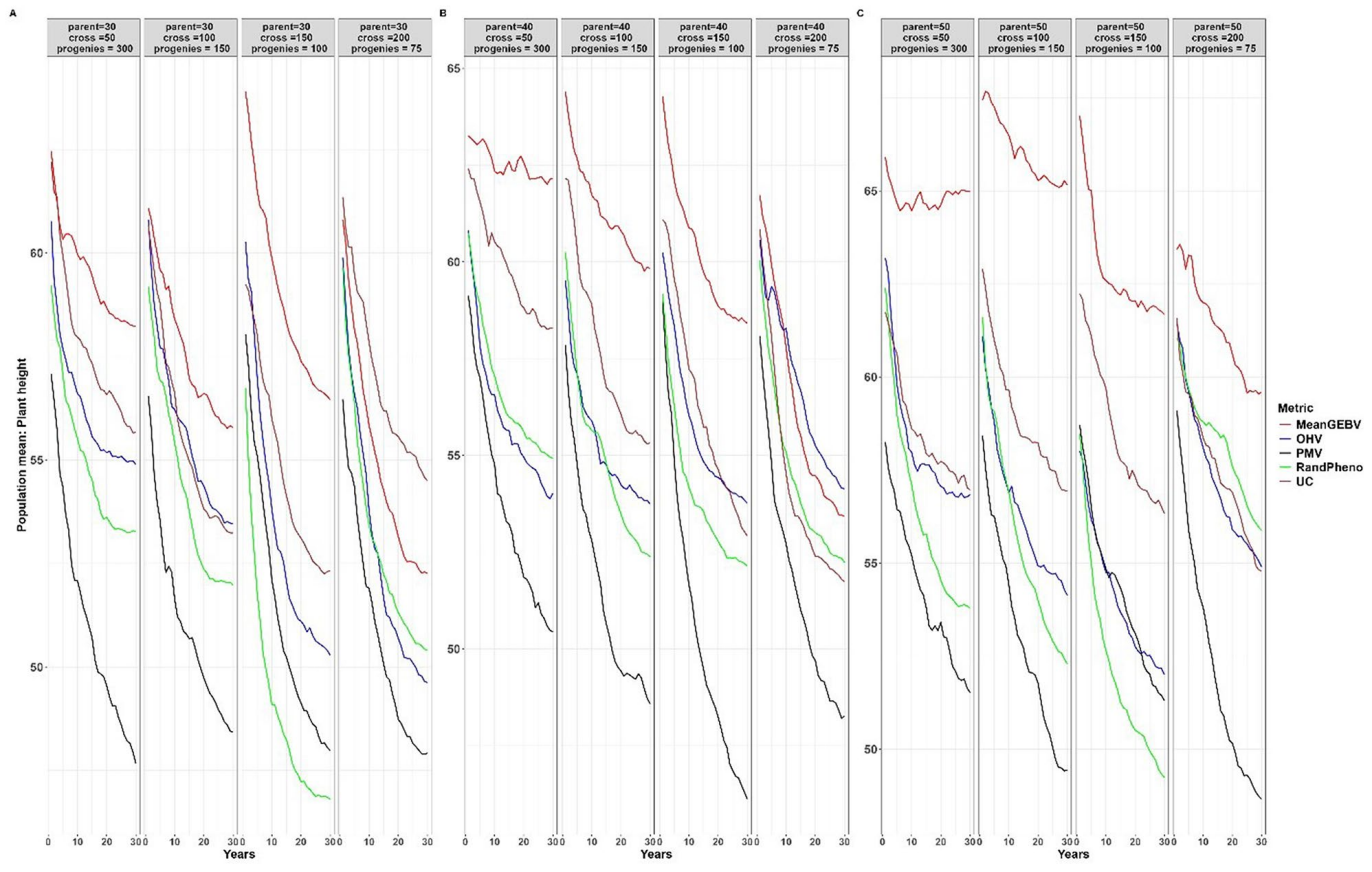
Population mean for different cross-selection methods and different numbers of parents, crosses and progeny per cross for plant height over 30 years post burn-in. The red line (MeanGEBV) highlights the genetic gain obtained using the mean of the GEBV of the distantly related superior parents to select crosses, the green line (RandPheno) represents the random mating of the superior genotypes, the blue line (OHV) is the optimal haploid value, the black line (PMV) represents the posterior mean variance and the brown line (UC) represents the genetic gain observed using the usefulness criterion as a cross-selection metric

**Fig. 5:**
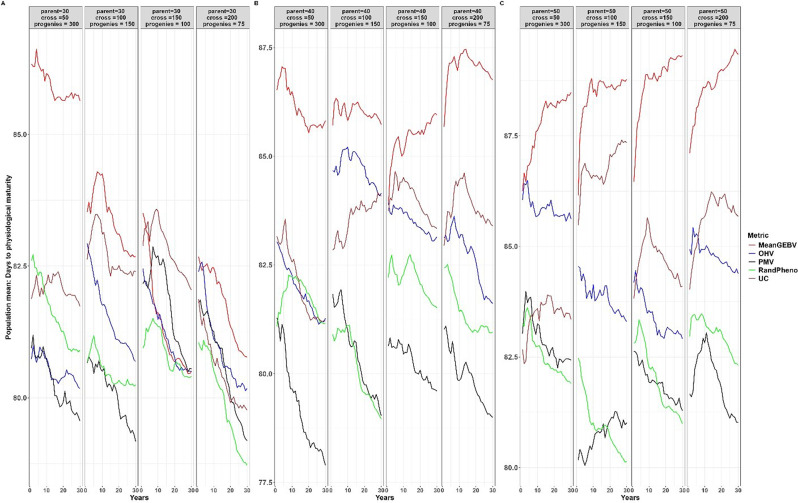
Population mean for different cross-selection methods and different numbers of parents, crosses and progeny per cross for days to physiological maturity over 30 years post burn-in. The red line (MeanGEBV) highlights the genetic gain obtained using the mean of the GEBV of the distantly related superior parents to select crosses, the green line (RandPheno) represents the random mating of the superior genotypes, the blue line (OHV) is the optimal haploid value, the black line (PMV) represents the posterior mean variance and the brown line (UC) represents the genetic gain observed using the usefulness criterion as a cross-selection metric

When compared to other cross-selection methods, the use of PMV as a cross-selection method consistently results in a high genetic gain or response to selection for traits where an increase is expected, such as YLD and TKW (Figs. 2 and 3). Additionally, it effectively facilitates the desired selection response for other traits, such as optimal PH and DPM (Figs. 4 and 5). Except when using 30 parents, 200 crosses, and 75 progeny per cross, UC showed marginal gains over PMV in the medium term (15 to 20 years post burn-in) for YLD. A similar trend was observed when superior parents were randomly mated in a breeding scenario that involved 50 crosses derived from 50 parents, each with 300 progeny per cross, for both YLD (Fig. 2) and TKW (Fig. 3).

In general, PMV outperforms other methods across the different breeding strategies evaluated in this study. For instance, employing PMV as the selection method, involving 40 parents, 50 crosses, and 300 progeny per parent, resulted in a higher genetic gain of 0.34% in the short term (1 to 10 years post-burn-in) and 8.56% in the long term (20 to 30 years post-burn-in) for YLD compared to gains achieved through random mating (Fig. 2). Moreover, using the same selection strategy, the mean population for PH across the breeding cycles was 53.59 cm with PMV, compared to 56.65 cm achieved through random mating of the superior parents (Fig. 4). When compared to the base population mean of 67.00 cm, PMV efficiently selected parental combination with optimal PH while sustaining gains for other primary traits. Moreover, the mean population for DPM was 79.25 days for PMV and 81.75 days when parents were randomly mated (Fig. 5). In comparison to the base population mean of 81 days, PMV led to a genetic gain of 2.19%.

Furthermore, the genetic gain for YLD improved by 7.53% in the short term and 16.32% in the long term when the number of crosses increased from 50 to 100 and the number of progeny per cross was reduced to 150 (Fig. 2). The mean population for PH using PMV was 51.52 cm, compared to 54.89 cm using random mating (Fig. 4). Interestingly, the DPM averaged at 80.44 days for PMV as selection method and closely a 80.16 days for random mating (Fig. 5). A similar trend was observed for other breeding scenarios. We emphasize the mean population values for both PH and DPM for simplicity, as these traits are expected to have optimal values in the long term, in contrast to the mean population values of the base parents.

**Fig. 6:**
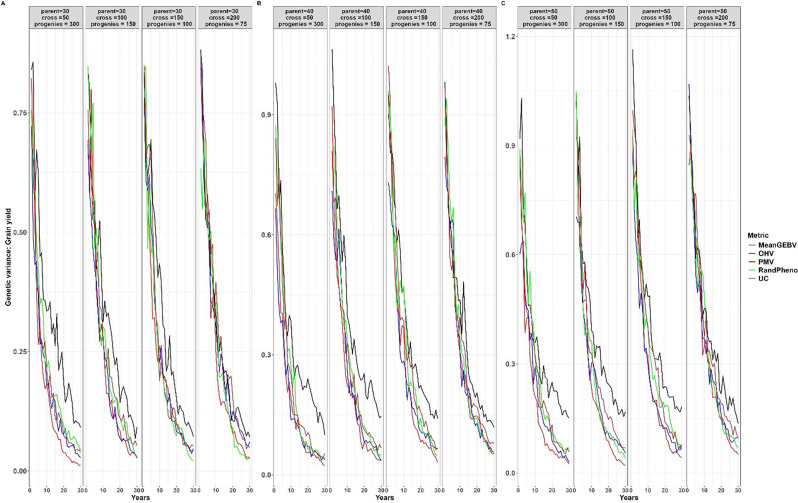
Genetic variance for different cross-selection metrics and different numbers of parents, crosses and progeny per cross for grain yield over 30 years post burn-in. The red line (MeanGEBV) highlights the genetic gain obtained using the mean of the GEBV of the distantly related superior parents to select crosses, the green line (RandPheno) represents the random mating of the superior genotypes, the blue line (OHV) is the optimal haploid value, the black line (PMV) represents the posterior mean variance and the brown line (UC) represents the genetic gain observed using the usefulness criterion as a cross-selection metric

In all breeding scenarios and for every trait we considered, PMV had higher genetic variance when compared to all other selection methods, as depicted in Fig. 6 and Supp 2:4. The magnitude of genetic variance loss per unit of time was lower with PMV when compared to our baseline method (RandPheno), especially in the medium and long term. For instance, using the smallest number of parents (30) and crosses (50) in our study, we observed a substantial reduction in the magnitude of genetic variance after 30 years post-burn-in. With random mating as the pairing method, the genetic variance diminishes to 0.02, 0.38, 0.10, and 0.22 for the traits YLD, TKW, PH, and DPM, respectively. However, when we employed PMV, the genetic variance remained notably higher, at 0.091, 1.69, 0.59, and 0.99 for the same set of traits. In general, PMV consistently shows greater genetic variance and a slower loss of diversity over time.

### Number of parents, number of crosses, and number of progeny per cross

**Fig. 7:**
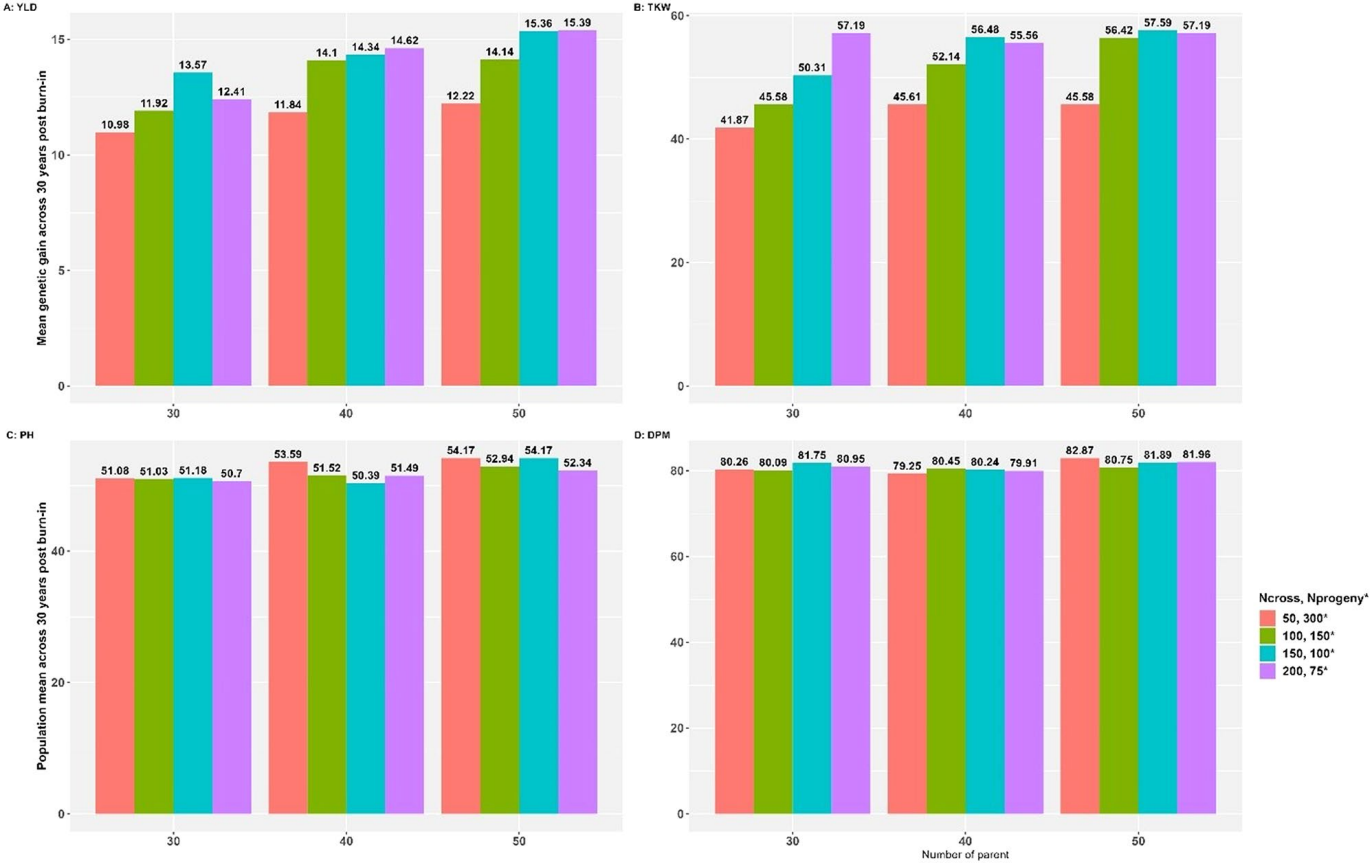
**A** and **B** represent genetic gains across 30 years post burn-in for grain yield (YLD) and 1000 kernel weight (TKW), and C and D represent the population mean across 30 years post burn-in for plant height (PH) and days to physiological maturity (DPM)

Genetic gain is influenced by a combination of factors (number of parents, crosses and progeny per cross) that appear to be interconnected, as depicted in Fig. 7. We selected the PMV for assessing the number of parents, crosses, and population size due to its superior efficiency when compared to other methods.

Increasing the number of crosses benefits from an increased number of parents but plateaus at 150 crosses (Fig. 7A:**D**). Considering YLD, the genetic gain values were 10.98, 11.92, 13.57, and 12.41 for 50, 100, 150, and 200 number of crosses and 300, 150, 100, and 75 number of progeny per cross using 30 number of parents (Fig. 7A**)**. The gain was only rapid when the number of crosses increased from 100 to 150. However, we observed diminishing returns when we further increased the number of crosses from 150 to 200. Similarly, when the number of parents was 40, the genetic gain increased from 11.84, 14.10, 14.34, and 14.62 for 50, 100, 150, and 200 crosses and 300, 150, 100, and 75 progeny per cross, respectively. Again, the gain from increasing the number of crosses beyond 150 was marginal. The same trend was observed with 50 parents, where the genetic gain increased from 12.22 for 50 crosses to 14.14 for 100 crosses, 15.36 for 150 crosses, and 15.39 for 200 crosses, while the number of progeny per cross remained at 300, 150, 100, and 75. Similar to the observation with 30 parents, there was no substantial improvement in genetic gain when increasing the number of crosses from 150 to 200.

Although there was a linear trend when the number of parents increased from 30 to 50, only marginal gains were observed when the number increased from 40 to 50. A similar trend was observed for TKW (Fig. 7B**)**, except that the linear increase in the number of crosses consistently improved the genetic gain, especially for 30 parents.

Considering PH, we found that with 30 parents, there was no significant difference in the mean population (15.08, 15.03, 51.18, and 50.70 cm) when increasing the number of crosses from 50 to 200 (Fig. 7C). However, it improved when compared to the mean population (67.00 cm) of the founder population. When we increased the number of parents from 40 to 50, we observed a similar pattern, except for a few differences. In the case of DPM, increasing the number of parents and the number of crosses did not translate linearly to an improved response to selection (Fig. 7D).

## Discussion

One of the key drawbacks of genomic selection is the loss of genetic variance when compared to conventional phenotypic selection in the long term [15]. In our study, we used stochastic simulation to evaluate the effectiveness of different genomic prediction cross-selection methods to predict the usefulness or merit of a cross, particularly when aiming for simultaneous improvement across multiple traits. Furthermore, this study aimed to support the NDSU pulse crop breeding program in determining the optimal number of parents, crosses, and progeny per cross, taking into account the constraints posed by the current breeding budget and logistical considerations.

The observed selection gain for all traits in various breeding scenarios and the cross-selection methods demonstrate the potential utility of using index selection as a derived phenotype within a genomic prediction framework. This approach is particularly valuable in situations where fitting multiple traits simultaneously might be computationally intensive or statistically challenging. Moreover, during the early yield testing stage, selecting for negatively correlated traits presents a significant challenge for weighted selection index methods, as optimal selection requires balancing trade-offs between traits. Additionally, RSI does not require estimates of genetic variances, covariances, or economic weights, which can be difficult to obtain accurately, especially in early-stage yield testing or with limited data.

In principle, RSI allows the selection of optimal parent combinations that have a balance of targeted multiple traits, taking into account their relative importance. Following a different rationale than the one described in our study, Chung & Liao [39] and Wolfe et al. [40] also reported an increase in genetic gain for simultaneous improvement of multiple traits using genomic prediction to predict the merit of crosses. Our approach is not without its limitations; its effectiveness may vary with different index selection methods. This variability could result in the inability to identify optimal breeding parents that effectively balance multiple traits relative to their importance. Additionally, the numerical sensitivity of some selection index methods can lead to transformations or scaling of traits, potentially affecting their biological relevance.

Studies [21, 23, 24, 40, 54] have reported that PMV serves as an unbiased predictor of progeny variance within bi-parental populations. This was attributed to PMV considering the haplotype of the parents, estimates of marker effects, and estimates of recombination frequencies between marker loci [21, 24]. Souza & Sorrells [55] suggested that the genetic gain achieved from a cross depends on the genetic variance of selected elite parents. Therefore, crosses with large genetic variance from the elite pool would theoretically generate a population with a favorable mean and contribute to increased genetic gain [7, 34, 54, 56]. This assumption will be invalid in a highly unstructured cross setting, where crossing parents is a mixture of elite and poorly performing lines. In practical breeding programs, the goal is often to maximize short-term gains while preserving long-term sustainability. Consequently, crosses between elite and poorly performing lines would be detrimental to achieving this objective [5, 34]. Our findings thus suggest a path to balance short-term gain and long-term sustainability.

In a simulation study by [26], they observed a decreasing predictive accuracy for progeny variance as the number of QTLs increased. In contrast, in our study, we did not observe a decrease in genetic gain for the traits we considered, despite variations in their genetic architecture. Furthermore, Lehermeier et al. [21] found no significant difference in the accuracy of progeny variance estimation when the number of QTLs was 300 or fewer. This difference in outcomes could be attributed, at least in part, to the method we used to estimate marker effects, which was based on index selection rather than individual traits. This strategy also addressed the challenge highlighted by [57]. They reported that crosses with extreme population means were accompanied by low genetic variance, while crosses with intermediate population means were associated with higher genetic variance. They explained that lines with similar genetic values will likely share alleles at the majority of quantitative trait loci (QTLs) underlying the trait, which accounts for the observed variation. However, this was not a concern in our proposed strategy because we are interested in predicting the variance of the index selection rather than individual traits, thus eliminating the chance of crossing poor lines with elite lines. For example, considering the smallest number of parents (30) and crosses (50) in our study, along with the increased genetic gain, PMV showed 4.56, 4.44, 5.90, and 4.50 times greater genetic variance and a slower rate of genetic variance for the traits YLD, TKW, PH, and DPM compared to the base method (random mating of the superior parents) after 30 years post burn-in.

The inconsistent genetic gain observed when the UC was used for selection decision might be due to the dependency of the UC on selection intensity and trait heritability. Unsurprisingly, Lehermeier et al. [21] found that the selection of crosses based on UC is more advantageous with increased selection intensity and high heritability. In our preliminary analysis (data not shown), we examined the performance of the UC method in comparison to PMV when considering single trait. Our preliminary result showed that PMV consistently outperformed UC, suggesting that the surrogate trait used did not adversely affect UC performance in our study.

Unexpectedly, OHV performed less favorably, and our results were consistent with previous studies [21, 58]. Theoretically, OHV assumes an infinite number of progeny per cross and selection intensity [21, 22], an assumption not met in our study. Furthermore, we did not fine-tune the number of segments where the absence of recombination is assumed, which is crucial for arriving at an optimal value.

Our results showed that the number of parents involved in crossing has a significant impact on both the population mean and genetic variance across the breeding cycles. In particular, when fewer parents were involved, we observed a slower rate of genetic improvement and an elevated risk of losing genetic variance. This is primarily attributed to the lack of unique crosses, especially with the increased number of crosses. Additionally, alleles that were lost as a result of limited parental diversity were not regained in subsequent generations. This leads to the observed rapid decline in genetic variance, particularly in the long term, resulting in reduced genetic gains compared to scenarios where a greater number of parents are involved.

Recently, Sabadin et al. [46] also emphasized the relationship between the number of parents and the effective population size (*N*_*e*_). In the simulation study, the author reported greater resilience to the loss of genetic variance over the long term, involving 48 parents compared to 24 parents. The decrease in genetic gain when few individuals are used to form the next generation suggests that the effect of genetic drift may far outweigh the effect of response to selection [34]. Therefore, selecting the appropriate number of parents for a breeding program is a pivotal factor for accelerating genetic progress, which directly impacts the program overall success [5, 59].

Considering the resource constraints on the breeding program, such as limitations on the number of lines that can be evaluated, it becomes crucial to identify the balance between maximizing genetic gain and preserving valuable genetic diversity. Generally, increasing the number of crosses enhances genetic gain and reduces the risk of genetic drift; however, there is not much gain to achieve much larger than increasing the crosses from 50 to 150 with a population size of 300 to 100 at any given number of parents. Similarly, Covarrubias-Pazaran et al. [59] also reported a sustained genetic gain in the long term with an increased number of crosses and fewer progeny per cross but with diminishing returns to additional crosses with fewer parents. Therefore, to achieve sustainable genetic progress in a breeding program, especially for small breeding programs, caution should be exercised when determining the optimal number of parents, crosses, and progeny per cross.

Despite these insights, our study did not investigate all possible combinations of the number of crosses, progeny per cross, and parents. This limit drawing definitive conclusions about the joint influence of these variables on genetic variance. Specifically, the inverse relationship between the number of crosses and the number of progeny per cross complicates understanding their effects on genetic variance. For instance, while increasing the number of crosses generally enhances genetic variance, decreasing the number of progeny per cross could counteract this effect due to genetic drift. Despite these limitations, our findings offer valuable insights, particularly for small breeding programs, and provide recommendations for the NDSU pulse breeding program on selecting the most suitable breeding strategy under constrained scenarios.

## Conclusion

We presented a simple but efficient approach to identify optimal crosses that simultaneously improve the genetic gain of multiple traits using index selection of the parents, parental haplotypes, marker effects, and recombination frequencies between marker loci. We proposed the use of this cross-selection strategy in a breeding program implementing GS to continuously sustain genetic improvement. For continued population improvement and the release of new varieties to the market, the use of genetic simulation to guide optimal resource allocations (number of parents, crosses and progeny per cross) and the design of crossing blocks is highly recommended. The underlying assumptions and simulated genetic parameters were tailored to the NDSU pulse breeding program, which might limit its generalization to other programs. To validate these results and extend it relevance to diverse breeding programs, empirical data should be used in multiple breeding programs. Nevertheless, our results serve as a guide for continuous genetic improvement in any public plant breeding program.

### Electronic supplementary material

Below is the link to the electronic supplementary material.

Supplementary Material 1

## Data Availability

No datasets were generated or analysed during the current study.
