# Supplementary material for: Genomic-inferred cross-selection methods for multi-trait improvement in a recurrent selection breeding program
Source: Plant Methods. 2024 Sep 2;20:133. doi: 10.1186/s13007-024-01258-4 (PMC11367796; doi:10.1186/s13007-024-01258-4)
Supplement: Supplementary file 1 — Supplementary Material 1 [file 13007_2024_1258_MOESM1_ESM.docx]

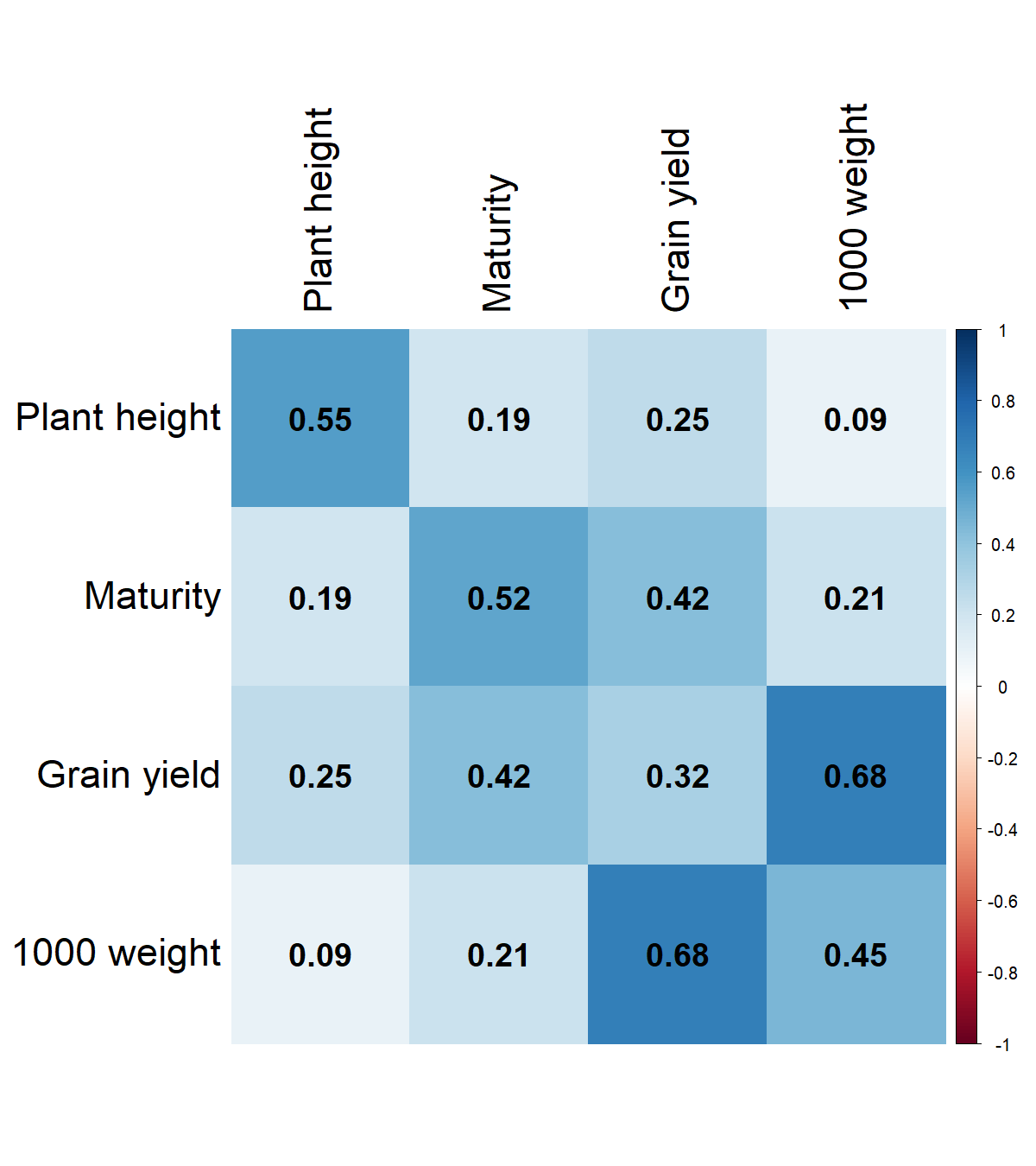


**Supplementary 1: Genetic correlation of grain yield, 1000 kernel weight, plant height and days to physiological maturity. The diagonal represents heritability for each trait.**


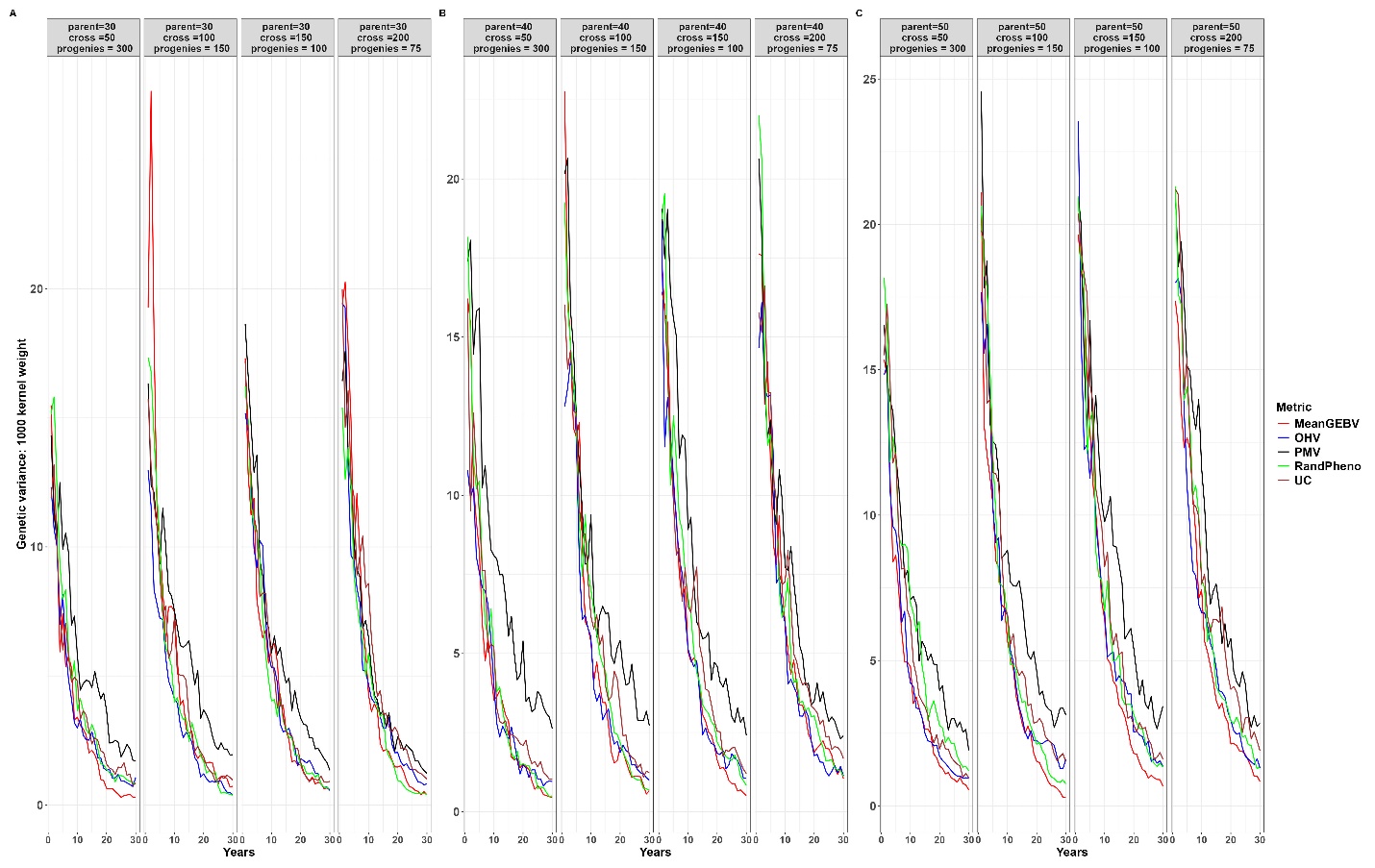


**Supplementary 2: Genetic variance for different cross-selection metrics and different numbers of parents, crosses and progeny per cross for 1000 kernel weight over 30 years post burn-in. The red line (MeanGEBV) highlights the genetic gain obtained using the mean of the GEBV of the distantly related superior parents to select crosses, the green line (RandPheno) represents the random mating of the superior genotypes, the blue line (OHV) is the optimal haploid value, the black line (PMV) represents the posterior mean variance and the brown line (UC) represents the genetic gain observed using the usefulness criterion as a cross-selection metric.**


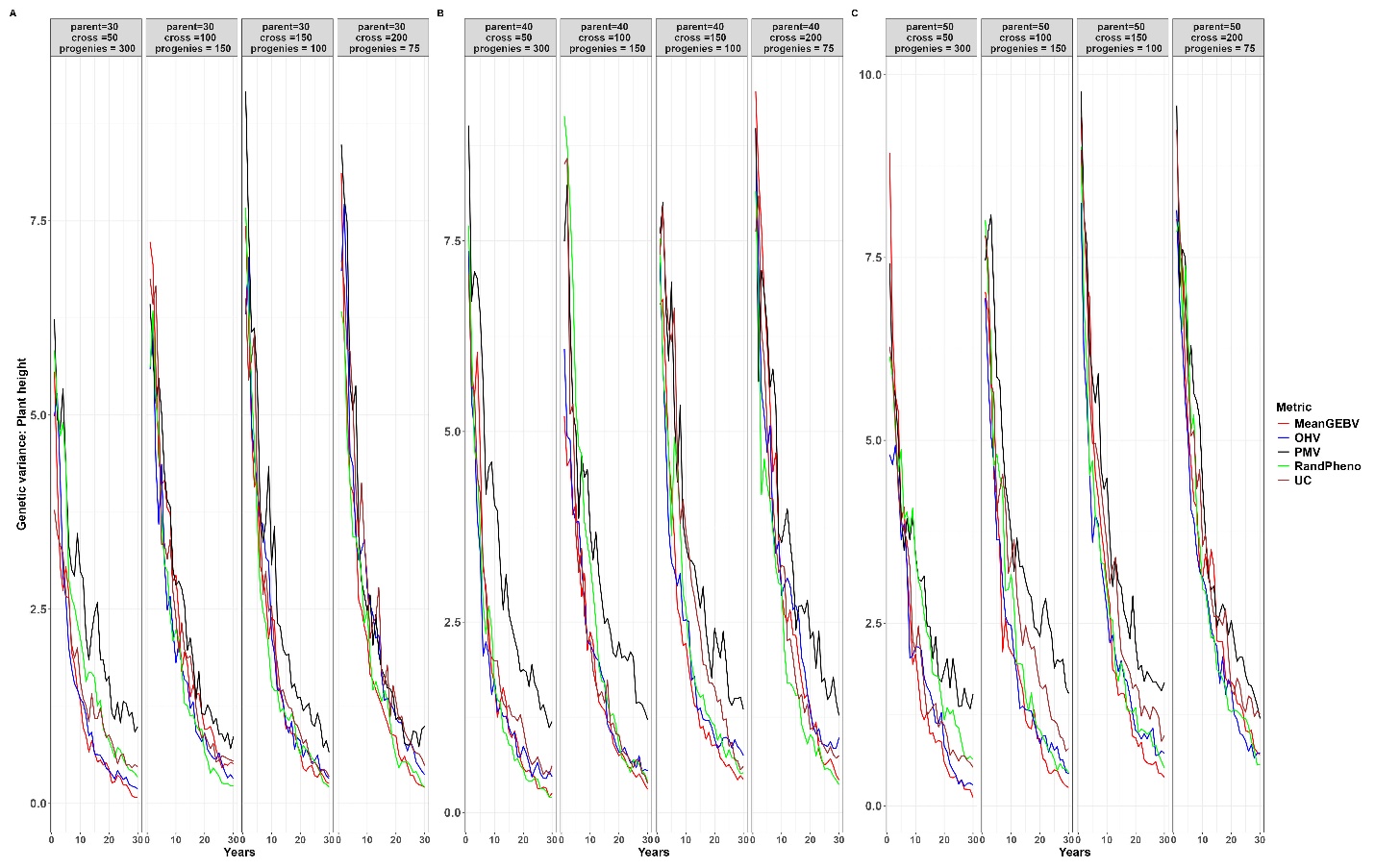


**Supplementary 3: Genetic variance for different cross-selection metrics and different numbers of parents, crosses and progeny per cross for plant height over 30 years post burn-in. The red line (MeanGEBV) highlights the genetic gain obtained using the mean of the GEBV of the distantly related superior parents to select crosses, the green line (RandPheno) represents the random mating of the superior genotypes, the blue line (OHV) is the optimal haploid value, the black line (PMV) represents the posterior mean variance and the brown line (UC) represents the genetic gain observed using the usefulness criterion as a cross-selection metric.**


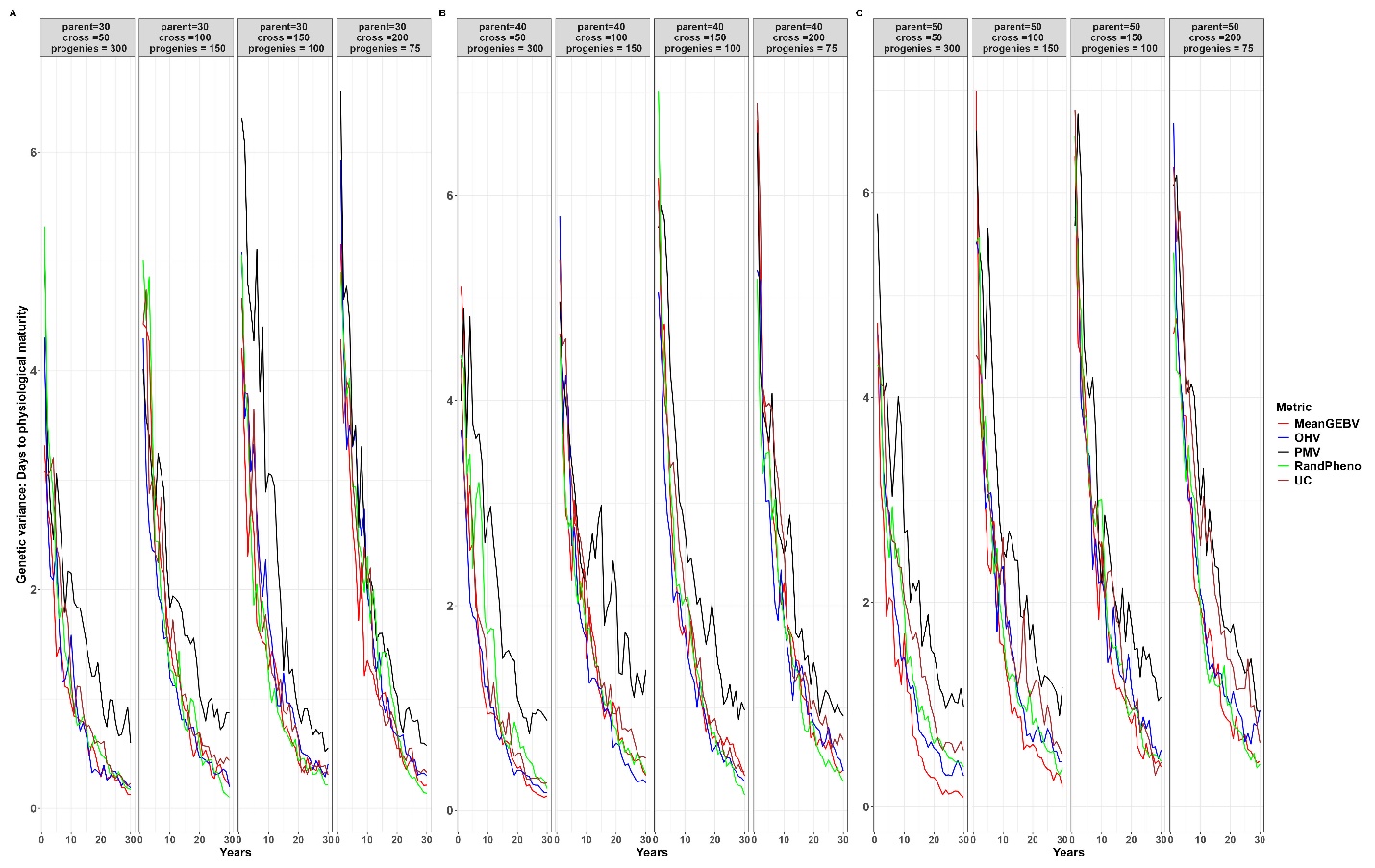


**Supplementary 4: Genetic variance for different cross-selection metrics and different numbers of parents, crosses and progeny per cross for days to physiological maturity over 30 years post burn-in. The red line (MeanGEBV) highlights the genetic gain obtained using the mean of the GEBV of the distantly related superior parents to select crosses, the green line (RandPheno) represents the random mating of the superior genotypes, the blue line (OHV) is the optimal haploid value, the black line (PMV) represents the posterior mean variance and the brown line (UC) represents the genetic gain observed using the usefulness criterion as a cross-selection metric.**
